# Editorial to the Special Issue SELSA: “Sensors for Environmental and Life Science Applications”

**DOI:** 10.3390/s21165353

**Published:** 2021-08-09

**Authors:** Najla Fourati, Mohamed M. Chehimi

**Affiliations:** 1 SATIE Laboratory, UMR CNRS 8029, Conservatoire National des Arts et Métiers, 75003 Paris, France; 2 ITODYS, Université de Paris & CNRS (UMR 7086), 75013 Paris, France

“Warn, inform, and prevent” are three essential elements to remember when designing sensors for real-time and in situ monitoring of organic, inorganic, and macromolecular compounds as well as micro-nanoparticles and microorganisms. Within the spirit of analytical sciences, we wished to gather young researchers, early academics, and experts to share their knowledge of building highly sensitive and selective sensors for environmental and life sciences. Indeed, chemo- and biosensors are the most appropriate devices that can fulfil the requirements of monitoring a variety of relevant species (molecules, ions, enzymes, particles, cells). In addition, time and effort have been spent on the development of electrochemical, optical, and gravimetric sensors, among others, directed towards the minimal preparation of sensing materials with sub-picomolar detection, portability for on-site and wireless/remote detections, and multiplexed and/or parallel sensing.

We were delighted to receive fourteen contributions from very enthusiastic colleagues, spread over two letters, eight original papers and four reviews. Eighty-one authors, from fifteen countries and four continents, contributed to SELSA. They have shared the very best of their recent original findings or summarized the state-of-the-art chemosensors and biosensors in authoritative reviews.

Developing any kind of sensor requires the understanding of sensor–analyte interactions at the molecular level. In this regard, computational chemistry has been demonstrated to be a fantastic tool that the sensor experimentalist could use to understand, for example, how highly toxic gases interact with pristine and functionalized graphene in view of developing highly sensitive and selective sensors of gases that contribute to global warming, ozone depletion, acid rain, and climate change [1].

Three reviews point out the role of electrochemical sensors for water quality monitoring [2] with nanostructured highly sensing layers. It has been demonstrated that polymer imprinting technology enhances selectivity of the electrochemical and impedimetric sensors of metal ions, Cr(VI), pesticides, and bacteria [2,3,4]. In the domain of electrochemical sensors, much hope is now placed on carbon paste electrodes (CPEs) for their ease of preparation, high surface area, and versatility. CPEs have been employed for making potentiometric sensors for monitoring total residual chlorine in electrolytically-treated ballast water (BW) used to ensure the stability of ships [5]. This letter draws our attention to a topic of major importance pertaining to the ever-growing ocean transportation in world trade and the associated pollution. In another letter, imprinting technology is demonstrated to advantageously boost the optical detection of protein using birefringent liquid crystals [6].

SELSA SI features eight original contributions that emphasize the role of mathematical modelling intended to decrease measurement time, which is absolutely necessary for the real-time detection of different analytes present in food [7], or to optimize the design of gravimetric, acoustic wave transducers [8]. Sensing performances depend on the understanding of each single physicochemical parameter while performing sensing measurements, e.g., pH and ionic strength [9]. However, besides these essential aspects, it is to note that single-signal output could be affected by background noise or electrode making, or complexity of the matrix under test, which may induce low accuracy. To address this important issue, ratiometric strategy based on dual-signal output is featured and recommended to build sensors with inner correction [10]. Other improvements in electrochemical sensors consist of designing electrode arrays and their use for sampled-current voltammetry coupled to anodic stripping voltammetry. This approach is suitable for miniaturization and a 300-fold output signal could be obtained compared to linear sweep anodic stripping voltammetry [11].

Applied sensor science and technology aspects report the development of infrared-based optical sensors of particulate matter with an emphasis on chemical composition, in contrast to state-of-the-art sensors focusing only on particle size, shape, and concentration [12].

Finally, carbon allotropes continue to fascinate sensor specialists keen on implementing these nanomaterials in view of enhancing selectivity and/or sensitivity; the question is still open as to whether carbon nanotubes [13] or graphene nanosheets [14] should be used in this regard.

From the above, we do hope that SELSA SI will be of great interest to the readership of the well-known and esteemed MDPI journal Sensors. Our contributors have provided guidance and guidelines for crafting numerous sensors relevant to life and environmental sciences. We anticipate that SELSA SI will be exciting reading to the specialist and non-specialist alike, i.e., students, academic researchers, and start-up and industrial engineers.

To finish, we are very grateful to our contributors and the professional team at MDPI who have constantly assisted us during the whole process of SELSA.

From the above, we do hope that SELSA SI will be of great interest to the readership of the well-known and esteemed MDPI journal Sensors.

## Data Availability

Not applicable.
